# EMPTY PERICARP11 serves as a factor for splicing of mitochondrial *nad1* intron and is required to ensure proper seed development in maize

**DOI:** 10.1093/jxb/erx212

**Published:** 2017-09-30

**Authors:** Xuemei Ren, Zhenyuan Pan, Hailiang Zhao, Junli Zhao, Manjun Cai, Jiang Li, Zuxin Zhang, Fazhan Qiu

**Affiliations:** National Key Laboratory of Crop Genetic Improvement, Huazhong Agricultural University, Wuhan, P.R. China

**Keywords:** alternative oxidase, Complex I assembly, embryo, endosperm, NADH dehydrogenase, ultrastructure

## Abstract

Group II introns are common in the mitochondrial genome of higher plant species. The splicing of these introns is a complex process involving the synergistic action of multiple factors. However, few of these factors have been characterized in maize. In this study, we found that the *Empty pericarp11* (*Emp11*) gene, which encodes a P-type pentatricopeptide repeat (PPR) protein, is required for the development of maize seeds. The loss of *Emp11* function seriously impairs embryo and endosperm development, resulting in empty pericarp seeds in maize, and alteration in *Emp11* expression leads to quantitative variation in kernel size and weight. We found that the *emp11* mutants showed a failure in *nad1* intron splicing, exhibited a severe reduction in complex I assembly and activity, mitochondrial structure disturbances, and an increase in alternative oxidase *AOX2* and *AOX3* levels. Interestingly, the *emp11* phenotype was very severe in the W22 inbred line but could be partially recovered in B73 BC_2_F_2_ segregating ears. These results suggest that EMP11 serves as a factor for the splicing of mitochondrial *nad1* introns and is required for mitochondrial function to ensure proper seed development in maize.

## Teaser

A new pentatricopeptide repeat (PPR) gene in maize differs from previously reported genes, as it is required for splicing of all four introns of *nad1*.

## Introduction

Mitochondria are key players in plant development, fitness and reproduction. They perform a variety of fundamental functions, for example in pyruvate oxidation, the Krebs cycle, and the metabolism of amino acids, fatty acids, and steroids, with the most crucial being the production of ATP by oxidative phosphorylation (Mackenzie and McIntosh, 1999; Gualberto *et al.*, 2014). Although most mitochondrial proteins are encoded by nuclear loci, the mitochondrion is a semi-autonomous organelle with its own genome. The maize NB (Normal type in nuclear background B73) mitochondrial genome encodes 58 genes incorporating 22 introns, which all belong to group II introns based on their distinctive structures (Clifton *et al.*, 2004; Bonen, 2008). Group II intron splicing depends on various protein factors including maturases (MATs), which are encoded by group II introns in bacteria and in yeast (Singh *et al.*, 2002; Mohr and Lambowitz, 2003). The MATs can also be encoded by nuclear genomes of plant species, such as the four maturases, nMAT1–4, in Arabidopsis. (Mohr and Lambowitz, 2003; Keren *et al.*, 2009; Keren *et al.*, 2012; Cohen *et al.*, 2014).

Pentatricopeptide repeat (PPR) proteins, a large protein family in plant species, also play constitutive and essential roles in the diverse processes of organelle RNA processing, including RNA editing, cleavage, splicing, and stability (Lurin *et al.*, 2004; Schmitz-Linneweber and Small, 2008; Fujii and Small, 2011; Barkan and Small, 2014). Mutations in PPR genes result in distinct physiological and morphological defects, indicating the importance of PPR proteins for plant growth and development, especially for embryo and endosperm development (Barkan and Small, 2014). For example, OTP43, a P-type PPR protein, is required for *trans*-splicing of the mitochondrial *nad1* intron 1 in Arabidopsis. *otp43* mutants show severe defects in seed development, germination, and plant growth (Falcon de Longevialle *et al.*, 2007). Four P-type PPR proteins have been reported to be involved in the splicing of mitochondrial genes in maize. EMPTY PERICARP16 is required for mitochondrial *nad2* intron 4 *cis*-splicing in maize (Xiu *et al.*, 2016). Plants with mutations in *EMP16* show an empty pericarp phenotype, reduced complex I assembly and activity, increased accumulation of complex III, and increased expression of alternative oxidase *AOX2* (Xiu *et al.*, 2016). DEK35 is required for the *cis*-splicing of mitochondrial *nad4* intron 1. The *dek35* mutant exhibits impaired mitochondrial structure and delayed seed development (Chen *et al.*, 2016). A third PPR protein, Dek2 targets mitochondria as well; the *dek2* mutation reduces the splicing efficiency of mitochondrial *nad1* intron 1 and also leads to small kernels (Qi *et al.*, 2016). Recently, EMP10 was found to be involved in the splicing of mitochondrial genes, which affects the *cis*-splicing of *nad2* intron 1 and seed development in maize (Cai *et al.*, 2017). Although the PPR family has many members, there seems to be little redundancy between different family members (Barkan and Small, 2014). The maize genome encodes hundreds of PPR proteins, however, the fundamental molecular functions of the majority of PPR proteins are still unknown. Here, we report the biological functions of the P-type PPR gene *Emp11* in maize. *Emp11* is required for the splicing of all *nad1* introns. The loss of *Emp11* function leads to a severe reduction in complex I assembly and activity and an increase in alternative oxidase *AOX2* and *AOX3* levels. The *emp11* mutants are arrested in both embryo and endosperm development, resulting in empty preicarp seeds, but the *emp* phenotype can be partially recovered in B73 BC_2_F_2_ segregating ears. In summary, we present a new PPR protein that differs from previously reported genes by being required for splicing of all *nad1* introns in maize.

## Materials and methods

### Plant materials

The *emp11-1* and *emp11-2* mutants were isolated from two UniformMu lines requested from the UniformMu collection stocks (McCarty *et al.*, 2005): UFMu-08197 and UFMu-04323, respectively. The two UniformMu lines were backcrossed to the W22 inbred line into BC_2_. Mutator insertion loci were screened using gene-specific primers and Mutator-specific primers (McCarty *et al.*, 2005). Normally developed kernels in a segregating ear were randomly planted and the kernel phenotype of each progeny individual was then evaluated. Meanwhile, total DNA was extracted from a leaf of each individual and each kernel with the pericarp removed in the segregating ear, using a modified CTAB method (Shahzadi *et al.*, 2010), and was then used for genotype analysis and co-segregation analysis. In addition, *emp11-1* heterozygous (+/*emp11-1*) individuals on the W22 background were crossed to the B73 line to develop a BC_2_F_2_ population where the *+*/*emp11-1* and *emp11-1/emp11-1* genotypes were selected and referred to as *+*/*emp11-1* (B73) and *emp11-1/emp11-1* (B73).

### Cytological observation

Kernels at 6, 8, 12 and 15 d after pollination (DAP) were collected and then cut along the longitudinal axis. The cut kernels were fixed overnight in 4% paraformaldehyde (Sigma, Santa Clara, CA, USA), dehydrated in an ethanol gradient series (30, 50, 70, 85, 95, and 100% ethanol), and embedded in Paraplast Plus (Sigma, Santa Clara, CA, USA). The sample blocks were sectioned into 8 µm slices using a Leica RM2265 microtome (Leica Microsystems, Wetzlar, Hesse-Darmstadt, Germany) and stained with 0.5% toluidine blue O. Images were captured using a Leica MZFLIII microscope (Leica Microsystems, Wetzlar, Hesse-Darmstadt, Germany). Additionally, for TEM analysis, endosperms at 10 DAP were cut into 1 mm^3^ pieces. Fresh tissues were fixed overnight in 2.5% (w/v) glutaraldehyde in 0.1 M phosphate buffer at pH 7.4, fixed in 2% OsO4 in the same buffer, and then dehydrated and embedded in epoxy resin and SPI-812 (Structure Probe, Inc., West Chester, PA, USA). Ultra-thin sections obtained using a Leica UC6 ultra microtome (Leica Microsystems, Wetzlar, Hesse-Darmstadt, Germany) were stained with uranyl acetate and subsequently with lead citrate. The observations and recording of images were performed using a Hitachi H-7650 transmission electron microscope (HITACHI, Chiyoda-Ku, Tokyo, Japan) at 80 kV and a Gatan 832 CCD camera (Gatan, Pleasanton, CA, USA). The procedures were performed as described by Yi *et al.* (2010).

### RNA extraction and gene expression analysis

To analyze the expression of *Emp11*, the developing tissues, including the root, stem, leaf, tassel, ear, silk, ovary, endosperm, and embryo were collected. Total RNA was extracted from plant tissues using Ambion Pure Link Plant RNA Reagent (Life Technologies, Invitrogen, Carlsbad, CA, USA) and was then reverse transcribed using M-MLV reverse transcriptase (Invitrogen, Carlsbad, CA, USA) according to the manufacturer’s instructions. Reverse transcription PCR (RT-PCR) was performed for expression pattern analysis of *Emp11* with primer pairs RT-Emp11-3F and RT-Emp11-3F. Quantitative real time PCR (qRT-PCR) was completed using SYBR Select Master kit (Life Technologies, Invitrogen, Carlsbad, CA, USA) according to the manufacturer’s instructions with three biological replicates, and the maize actin gene (GRMZM2G126010) was used as the internal control. The relative expression levels were calculated using the comparative Ct method (Livak and Schmittgen, 2001). For mitochondrial and *AOX* gene expression, total RNAs were extracted from 15 DAP kernels of wild-type and two *emp11* mutant alleles with the pericarp removed. The RNAs were treated with RNase-free DNase and then normalized against both total RNA and *ZmActin* (GRMZM2G126010). The primers used for mitochondrial gene RT-PCR (Fig. 4A and Supplementary Fig. S6 at *JXB* online) were reported by Xiu *et al.* (2016). All the primers were anchored to the 5ʹ-UTR and 3ʹ-UTR, near the ATG and stop codon, of every gene in order to test the full-length coding region.

### Rapid Amplification of cDNA Ends (RACE)

RACE was performed with the SMART RACE cDNA amplification kit (BD Biosciences Clontech, Franklin Lakes, NJ, USA) according to the recommended protocol. Reactions for 3ʹ- RACE were conducted using primer F69 and nested primer F206. For the 5ʹ- RACE reactions, the universal primer provided with the kit was used in combination with primer R468 and nested primer R565. Primer sequences are listed in Supplementary Table S1.

### RNA *in situ* hybridization

mRNA in situ hybridization was performed on developing kernels at 10 DAP; kernels were trimmed along the medial-lateral axis and immediately fixed in 4% paraformaldehyde, following the procedure for cytological observation. Slides were deparaffinized and treated with 10 mg m^-1^ proteinase K. *In vitro* transcription of the digoxigenin-UTP-labeled (Roche, Basel, Basel-Stadt, Switzerland) probe was completed. RNA sense and antisense probes were obtained using T7 and SP6 polymerases. The probe used to detect the *Emp11* transcript corresponds to a 467 bp fragment from +656 to +1123bp of *Emp11* cDNA and was constructed using the RT-Emp11-3F/3R primers. The primer sequences are listed in Supplementary Table S1. The hybridization was performed at 42°C overnight in 50% formamide buffer containing 0.5 ng ml^-1^ kb^-1^ probe, 0.5 mg ml^-1^ yeast tRNA, 10% dextran sulfate, 20 mM NaCl, 10 mM NaH_2_PO_4_, and 1X Denhardt’s solution. The slide was washed with 50% formamide in 2X saline sodium citrate (SSC) buffer solution. After RNase A treatment, a high stringency wash was performed twice using 2X SSC and twice using 0.2X SSC, as described in Shen *et al.* (2013). Digoxigenin detection and signal visualization were completed using nitroblue tetrazolium and 5-bromo-4-chloro-3-indolyl phosphate (Roche, Basel, Basel-Stadt, Switzerland) according to the manufacturer’s instructions. Images were captured using a Nikon Eclipse 80i differential interference contrast microscope (Nikon, Chiyoda-Ku, Tokyo, Japan).

### Subcellular localization of EMP11

Subcellular localization was performed as described by Liu *et al.* (2013). The full-length coding sequence of *Emp11*without the termination codon was amplified using the Emp11-Kpn1-F and Emp11-Xba1-R primers, which are listed in Supplementary Table S1. The PCR product was purified and sequenced. The purified product with the correct sequence was then inserted into the pM999-GFP vector to generate a GFP fusion construct. The fusion construct was introduced into maize protoplasts from seedling leaves with polyethylene glycol (PEG)/calcium-mediated transformation (Yoo *et al.*, 2007). MitoTracker Red (Thermo Fisher Scientific, Waltham, MA, USA) was used to label the mitochondria.

### Mitochondrial complex activity assay

Crude mitochondria were isolated from the *emp11* mutants and the wild-type kernels at 12 DAP. The isolation of crude and intact mitochondria, blue native PAGE, and the in-gel complex I activity assay were performed as described (Meyer *et al.*, 2009; Keren *et al.*, 2012). Kernels with the pericarp removed at 12 DAP were ground at 4°C in extraction buffer comprising 0.3 M sucrose, 5 mM tetrasodium pyrophosphate (Sigma, Santa Clara, CA, USA), 10 mM KH_2_PO_4_ at pH 7.5, 2 mM EDTA, 1% [w/v] polyvinylpyrrolidone 40, 1% [w/v] BSA, 5 mM cysteine, and 20 mM ascorbic acid, using a Polytron or porcelain mortar and pestle. The extract was filtered through two layers of cheesecloth and two layers of Miracloth (Calbiochem Co., La Jolla, CA, USA). The homogenate was centrifuged for 5 min at 3 000 g. Mitochondria were obtained by centrifugation of the clear supernatant at 20 000 g for 10 min. The pellet was resuspended in wash buffer comprising 0.3 M sucrose, 1 mM EGTA, and 10 mM MOPS/KOH at pH 7.2, and subjected to the same low speed (3 000 g) and high speed (20 000 g) centrifugations. Mitochondrial proteins weighing 200–500 µg, were solubilized with dodecyl maltoside (Sigma, Santa Clara, CA, USA), 1–2% [w/v] final or 4% digitonin (Sigma, Santa Clara, CA, USA) in amino caproic acid (ACA) buffer comprising 750 mM amino caproic acid (Sigma, Santa Clara, CA, USA), 0.5 mM EDTA, and 50 mM Tris-HCl at pH 7.0, and incubated for 30 min at 4°C. The samples were centrifuged for 10 min at 20 000 g, and Serva Blue G (0.2% [v/v] final) was added to the supernatant as described in the manual. The samples were loaded onto a 3% to 12% gradient gel (Invitrogen, Carlsbad, CA, USA) with cathode buffer containing 0.02% dodecyl maltoside. For the in-gel activity assay, complex I was optionally analyzed on separate gel strips. Gel strips loaded with extracts from 200 µg of maize mitochondria were assayed for complex I activity in an assay buffer comprising 25 mg of nitrotetrazolium blue (Sigma, Santa Clara, CA, USA) and 100 µl of 10 mg/ml NADH (Sigma, Santa Clara, CA, USA) added to 10 ml of 5 mM Tris/HCl at pH 7.4.

### Maize transformation and progeny analysis

Antisense *Emp11* (GRMZM2G353301) was amplified with primers GM301-1F and GM301-1R (see Supplementary Table S1) and ligated into the binary vector pCAMIBA3300. The resulting RNAi vector, pZZ-GM301, was transformed into the maize ZZC01 line using Agrobacterium-mediated transformation. Genotypes were tested with the TransDNA-F/R primers, with the TransDNA-F primer designed for the *Emp11* sequence and the TransDNA-R primer designed for the vector sequence. Expression levels were tested with TransRNA-F/R primers designed for the 3ʹ-UTR of *Emp11*. The primers are listed in Supplementary Table S1.

## Results

### Phenotypic characterization of the *emp11-1* mutant

**Fig. 1.**
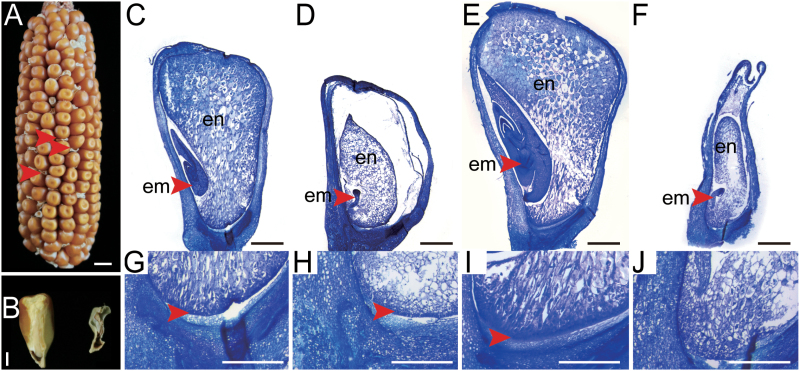
Embryo and endosperm development is arrested in *emp11-1* mutants. (A) The ears segregated 3:1 for wild-type and *emp11-1* mutant kernels (arrows). The *emp11-1* mutants were defective with an empty, collapsed, and wrinkled pericarp. (B) The embryo or endosperm structures of *emp11-1* kernels were abnormal at maturity. (C–J) Sections of developmental kernels at 12 DAP (C,D, G, H) and 15 DAP (E, F, I, J). Wild-type (C, E) and *emp11-1* kernels (D, F). (G–J) are magnified images of the micrographs above. Arrows indicate the BETL. em, embryo; en, endosperm. Scale bar, 1 cm in (A), 2 mm in (B), 1 mm in (C–F) and 500 µm in (G–J).

The *emp11-1* mutant was isolated from a UniformMu stock (No. UFMu-08197) with active Mutator (Mu) in the W22 genetic background (McCarty *et al.*, 2005). The *emp11-1* mutant exhibited empty pericarp seeds in mature maize ears (Fig. 1A). The mutant kernels could be clearly distinguished from their wild-type siblings at 6 DAP on the basis of their reduced size. At 12 DAP, the mutant kernels were dramatically different from wild-type kernels in a segregating ear, namely they were small, white, and shrunken. At maturity, the mutant kernels had almost no starch in the pericarp. During sectioning of the mature kernels, normal embryo or endosperm structures could not be found in the mutant kernels and they were non-viable (Fig. 1B). Homozygous mutant seedlings could therefore not be obtained.

To observe the development of the embryo and endosperm, *emp11-1* and wild-type kernels from a segregating ear were sectioned and examined (Fig. 1 and Supplementary Fig. S1). At 6 DAP, the embryos of *emp11-1* kernels were at the proembryo stage, similar to the wildtype, but the endosperm volume was dramatically smaller than wild-type kernels (Supplementary Fig. S1A, B). At 8 DAP, wild-type embryos had progressed from the proembyro to the coleoptile stage and endosperm cells filled the space inside the pericarp (Supplementary Fig. S1C), while *emp11-1* embryos remained at the proembyro stage, resembling 6 DAP embryos (Supplementary Fig. S1D), and *emp11-1* kernels were not filled by endosperm cells, with an obvious space between the pericarp and endosperm that persisted in later stages. At 12 DAP, wild-type embryos had a visible shoot apex (Fig. 1C) but *emp11-1* embryos were arrested at the transition stage (Fig. 1D). At 15 DAP, wild-type kernels had developed a nearly mature embryo and a starch-filled endosperm (Fig. 1E and Supplementary Fig. S1I) but *emp11-1* embryos remained arrested and the endosperm showed limited starch accumulation (Fig. 1F and Supplementary Fig. S1J). In summary, embryo and endosperm development were arrested in *emp11-1* mutants.

Moreover, an observation of the basal endosperm transfer layer (BETL) found that *emp11-1* kernels had a similar cellular morphology to wild-type kernels at early stages (Fig. 1G, H and Supplementary Fig. S1E–H). However, at 15 DAP, the BETL in *emp11-1* kernels was invisible (Fig. 1J). These results suggest that the *emp11-1* kernel defects may result from severe defects in development of the basal endosperm transfer cells.

### Characterization of the *Emp11* gene

**Fig. 2.**
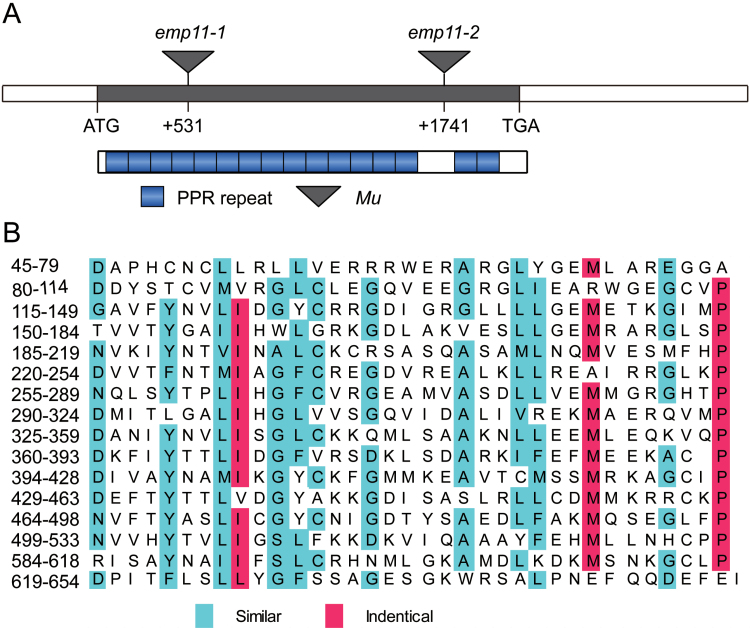
Characterization of the *Emp11* gene. (A) *Emp11* encodes a P-type PPR protein that contains 16 PPR repeats. Gene structure of *Emp11* and locations of the *Mu* insertions in two independent alleles. The *Mu* insertion sites in *emp11* alleles are indicated with triangles. (B) The 16 PPR motifs of the EMP11 protein are loosely conserved in alignment.

The *emp11-1* mutation behaved as a monogenic recessive trait since the mutant kernels on ears of self-pollinated heterozygous plants segregated in a ratio of 2235:800, wild-type: *emp11*, corresponding to a 3:1 segregation ratio (χ^2^-test, *P*>0.05; Fig. 1A). To identify the causal gene, the *Mu*-flanking sequences were identified using a method described by Settles *et al.* (2007). We found that a *Mu7* element was inserted into GRMZM2G353301 at +531 bp downstream of the predicted translation start site (ATG) in UFMu-08197 (Fig. 2A). A segregating F_2_ population was developed by self-crossing a heterozygous +/*emp11-1* plant. Genomic DNA was isolated from the individual F_2_ plants, and the phenotype was identified by selfed plants and checked for *emp11-1* segregation. As *emp11-1* homozygotes were not viable, only heterozygotes (segregating) or the wild-type (non-segregating) were available for this analysis. Importantly, the insertion site co-segregated with the phenotype in all the 901 individuals tested (+/+: +/*emp11-1*, 308:593, corresponding to 1:2 ratio, χ^2^-test, *P*>0.05) (Supplementary Fig. S2A, B).

To determine whether the mutation in GRMZM2G353301 was the cause for the *emp11* phenotype, we isolated an independent mutant, UFMu-04323, with a *Mu* element inserted at +1741 bp in the coding sequence (Fig. 2A) and we named this allele *emp11-2*. Importantly, the selfed progeny of +/*emp11-2* heterozygotes also produced empty pericarp kernels in a recessive pattern in a 3:1 ratio, with a wild-type: *emp11* ratio of 1516:497 (χ^2^-test, *P*>0.05) (Supplementary Fig. S2C, D). Complementation crosses between +/*emp11-1* and +/*emp11-2* produced mutant kernels as well with a wild-type: *emp11* ratio of 3896:1379 (χ^2^-test, *P*>0.05) (Supplementary Fig. S2E), confirming that GRMZM2G353301 is the causative gene for the empty pericarp phenotype.

To further explore the function of *Emp11*, RNAi transgenic plants were created in the maize inbred line ZZC01. It is likely that severe knockdown lines would be lethal considering the essential role of *Emp11* in W22, therefore we assume that only weak knockdown lines were recovered and two representative lines were used for further molecular analysis. qRT-PCR analysis revealed the *Emp11* expression level was significantly decreased in transgenic lines TG61 and TG68 compared with a non-transgenic control (NT) (Supplementary Fig. S3A). The T_1_ progeny of the two transgenic plants segregated defective kernels at different ratios, ~15% in line TG61 and ~22% in line TG68 (Supplementary Fig. S3B). The defective kernels were small, having 61% of the weight of the well-developed kernels in the TG61 line and 30% of the weight in the TG68 line (Supplementary Fig. S3D). These results showed that knockdown of *Emp11* expression affected kernel development and led to defective kernels, consistent with the UniformMualleles. Taken together, the results confirm that loss of GRMZM2G353301 is responsible for the *emp11* phenotype.

The genomic sequence and cDNA of *Emp11* were isolated from the W22 inbred line. Gene-specific primers were designed and used in rapid amplification of cDNA ends (RACE) to generate full-length wild-type *Emp11* cDNA (see Materials and Methods and Supplementary Table S1). *Emp11* was an intronless gene encoding a PPR protein with 698 amino acids, which were predicted to form 16 PPR motifs (Fig. 2A, B). The *emp11-1* allele contained a *Mu7* insertion in the fourth PPR motif-encoding region and *emp11-2* had a *Mu* insertion in the coding region between the fourteenth and fifteenth PPR motifs (Fig. 2A). EMP11 was most closely related to Sb05g002210 from *Sorghum bicolor* and LOC_Os11g04295 from rice (Supplementary Fig. S4).

### Expression and localization of *Emp11*

**Fig. 3.**
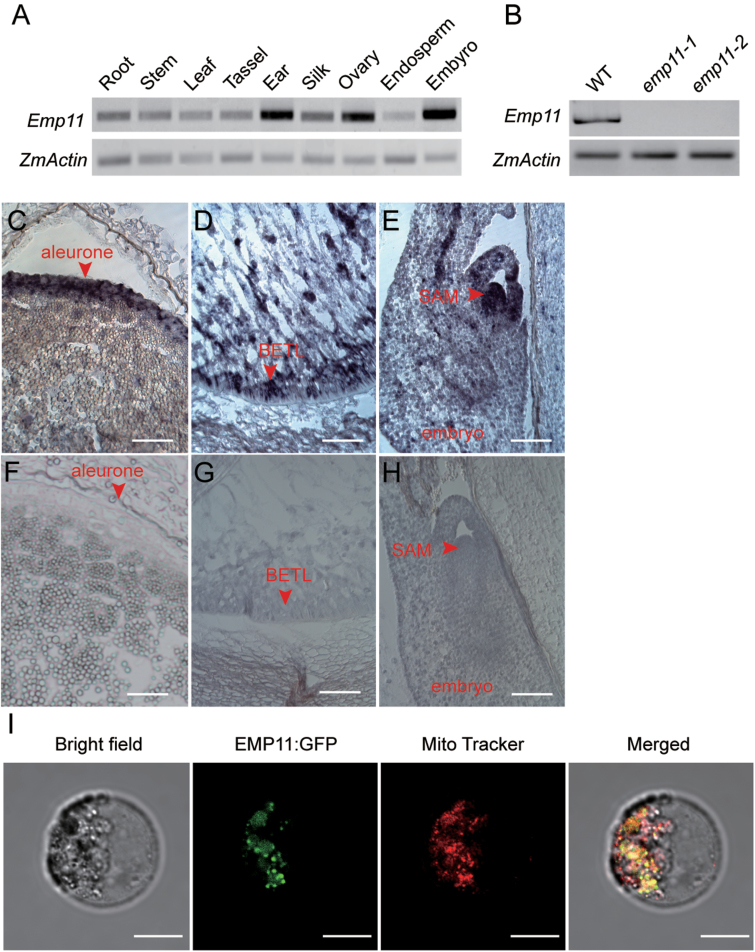
Expression and localization of *Emp11.* (A) RT-PCR analysis of *Emp11* expression in the root, stem, leaf, tassel, ear, silk, ovary, endosperm, and embryo 15 DAP. (B) RT-PCR analysis of *Emp11* expression in wild-type, *emp11-1*, and *emp11-2* seeds. RNA was isolated from kernels 15 DAP after pericarp removal. (C–F) RNA *in situ* hybridization was performed on developing kernels at 10 DAP using an *Emp11* antisense probe (C, D, E) and sense probe (F, G, H). Scale bar, 50 µm. (G) Subcellular localization of the EMP11 protein in mitochondria. The EMP11-GFP fusion protein was transiently expressed in maize leaf protoplasts and visualized with confocal microscopy. Scale bar, 10 µm.

To examine the expression pattern of *Emp11*, we performed RT-PCR and found that *Emp11* was expressed in all vegetative and reproductive tissues tested, with higher levels in the ear, ovary, and embryo and lower levels in the root, stem, leaf, tassel, silk, and endosperm (Fig. 3A and Supplementary Fig. S5). We next genotyped developing seeds with the pericarp removed. Homozygous *emp11* seeds with the pericarp removed were used to analyze expression of *Emp11* and mitochondrial genes. *Emp11* transcripts could not be detected in the *emp11-1* or *emp11-2* mutants (Fig. 3B), suggesting that both mutations were likely to be null. To understand the spatial localization of *Emp11*, mRNA *in situ* hybridization was performed in the wild-type kernels at 10 DAP. A strong hybridization signal was detected in the aleurone layer (Fig. 3C), the BETL (Fig. 3D) and in the embryo shoot apical meristem (Fig. 3E), with lower levels in other tissues, suggesting that *Emp11* was ubiquitously expressed but was higher in more metabolically active tissues. Furthermore, we characterized the subcellular localization of EMP11 using GFP fusion proteins. Transient expression of the EMP11-GFP fusion in maize protoplasts showed that GFP signal overlapped well with MitoTracker Red (Fig. 3I), demonstrating that EMP11 is targeted to mitochondria.

### Function of *Emp11* in mitochondrial *nad1* splicing

**Fig. 4.**
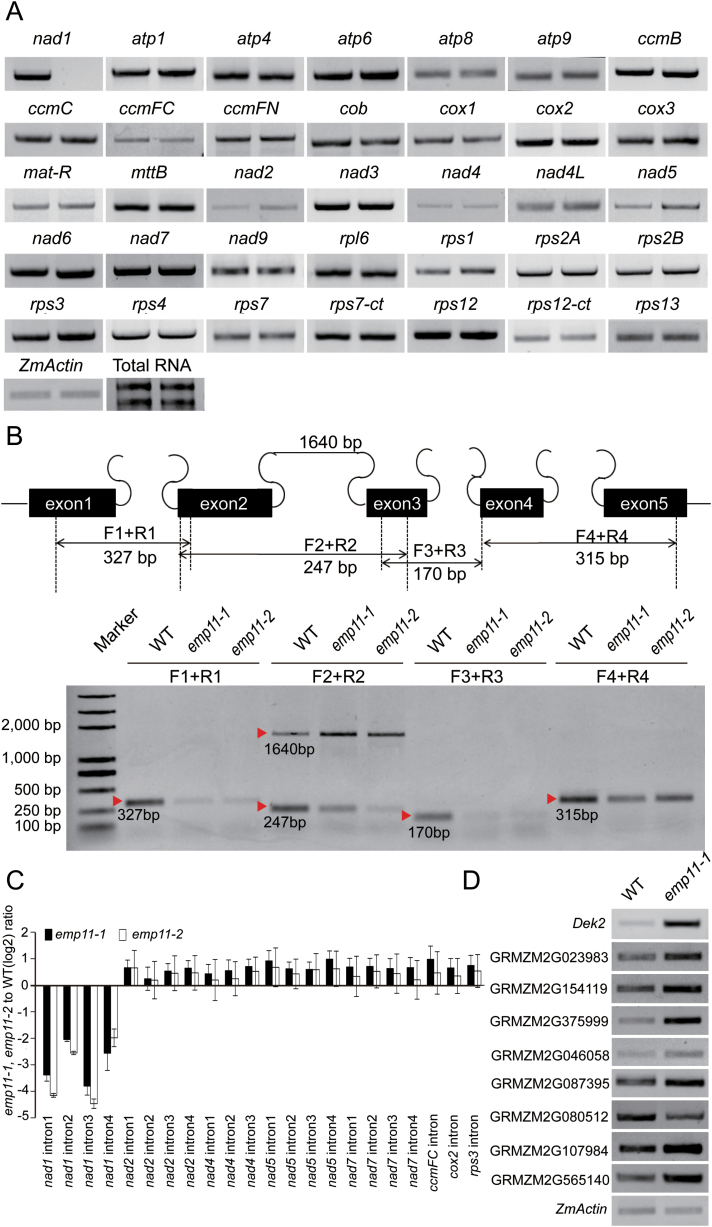
Deficiency of mature mitochondrial *nad1* transcripts in *emp11* mutants. (A) RT-PCR analysis of transcript levels of 35 mitochondrial-encoded genes in wild-type and *emp11-1* mutant plants. In each of the gels, the left lane is wild-type and the right lane is the *emp11-1* mutant. The RNA was isolated from the same ear segregating for wild-type and *emp11-1* mutants with the pericarp removed. (B) Structure of the maize mitochondrial *nad1* gene. Introns 1, 3, and 4 are *trans*-spliced introns and intron 2 is a *cis*-spliced intron. The *emp11* mutants were impaired in the splicing of *nad1* intron 1, 2, 3, and 4. The expected amplification products using different primer pairs are indicated. The RNA was isolated from the same ear segregating for wild-type and *emp11-1* mutants. (C) qRT-PCR analysis of splicing efficiency of all the 22 group II introns in *emp11-1, emp11-2*, and wild-type plants. The RNA was isolated from two mutant alleles and wild-type kernels at 15 DAP. Values represent the mean and standard deviation of three biological replicates. The expression levels were normalized to *ZmActin* (GRMZM2G126010). (D) RT-PCR analysis of *nad1* intron splicing-related genes in wild-type and *emp11-1*.

To investigate the function of *Emp11*, we analyzed the transcript levels of the maize NB mitochondrial genes in wild-type and *emp11-1* mutants by RT-PCR. Total RNAs were extracted from 15 DAP kernels of wild-type and *emp11-1* from the same segregating ear with the pericarp removed. The results showed that the expression level of most mitochondrial genes was not different between wild-type and the *emp11-1* mutant, but some mitochondrial tRNAs were more highly expressed in the *emp11-1* mutant (Fig. 4A and Supplementary Fig. S6A). Intriguingly, the fully spliced *nad1* transcript could not be detected in *emp11-1* kernels using primers targeted to the 5ʹ- and 3ʹ-UTRs, near the ATG and stop codon, but could be detected in wild-type kernels (Fig. 4A), suggesting that *Emp11* is potentially required for the regulation of *nad1* expression. The maize mitochondrial *nad1* gene contains three very long introns, introns 1, 3, and 4, which can’t be amplified by PCR and one 1393 bp intron, intron 2, which can be amplified using the F_2_+R_2_ primer pair shown in Fig. 4B (Brown *et al.*, 2014). To examine whether an intron-splicing deficiency leads to the absence of mature *nad1* transcript, we compared the transcripts for the presence of unspliced introns by RT-PCR. The results showed that intron1-unspliced transcripts were not detected in both wild-type and mutants, due to a failure in PCR amplification of the long intron1 using the F_1_+R_1_ primer pair, but levels of intron1-spliced PCR products, 327 bp in size, were lower in *emp11* mutants than in wild-type (Fig. 4B). Levels of intron3-spliced PCR products, 170 bp in size, and intron4-spliced PCR products, 315 bp in size, which were amplified using F_3_+R_3_ and F_4_+R_4_ primer pairs, respectively, were also lower in *emp11* mutants than in wild-type (Fig. 4B). Both intron 2-unspliced, 1640 bp in size, and intron 2-spliced, 247 bp in size, transcripts could be amplified using F_2_+R_2_ primer pairs, and levels of the intron 2-spliced transcripts were lower in *emp11* mutants than in wild-type, whereas levels of unspliced transcripts were higher in *emp11* (Fig. 4B). The splicing defects of *nad1* introns in *emp11* mutants were also revealed by qRT-PCR (Fig. 4C). These results demonstrated that *Emp11* was required for the splicing of all *nad1* introns. We also examined the splicing efficiency of other mitochondrial group II introns by RT-PCR (Supplementary Fig. S6B). Both the RT-PCR and qRT-PCR results showed that all of the other mitochondrial group II introns were spliced normally in *emp11*. These results confirm that *nad1* intron splicing is specifically defective in the *emp11* mutants.

In Arabidopsis, six nuclear-encoded factors are required for the excision of *nad1* introns, including OTP43 (Falcon de Longevialle *et al.*, 2007), nMAT1 (Keren *et al.*, 2012), and nMAT4 (Cohen *et al.*, 2014) for intron 1; nMAT2 (Keren *et al.*, 2009), mCSF1 (Zmudjak *et al.*, 2013), and PMH2 (Köhler *et al.*, 2010) for intron 2; and mCSF1, nMAT4, and PMH2 for intron 3. Only nMAT4 is involved in the splicing of *nad1* intron 4. The maize orthologs of *nMAT1*, *nMAT2, nMAT4*, *OTP43,* and *mCSF1* are GRMZM2G023983, GRMZM2G154119, GRMZM2G375999, GRMZM2G 046058, and GRMZM2G087395, respectively. *PMH2* orthologs are GRMZM2G080512, GRMZM2G107984, and GRMZM2G565140. In maize, Dek2 is involved in the splicing of mitochondrial *nad1,* since the *dek2* mutation reduces the splicing efficiency of *nad1* intron 1. To investigate the *nad1* splicing mechanism in maize, we assayed the expression of *dek2* and the eight maize orthologs genes in *emp11-1* and wild-type plants by RT-PCR. The results showed that the expression of some genes was significantly higher in *emp11-1* plants than in wild-type, for example, *dek2*, GRMZM2G375999, GRMZM2G107984, and GRMZM2G565140 (Fig. 4D). In Arabidopsis, *nad1* has two *trans*-splicing introns, introns 1 and 3, while in maize, *nad1* introns 1, 3, and 4 are *trans*-spliced. These results show that *nad1* splicing in maize likely shares a complex mechanism with Arabidopsis and the absence of *Emp11* induces a higher expression of *nad1* splicing-related genes.

### Mitochondrial structure and respiratory function in *emp11* mutants

**Fig. 5.**
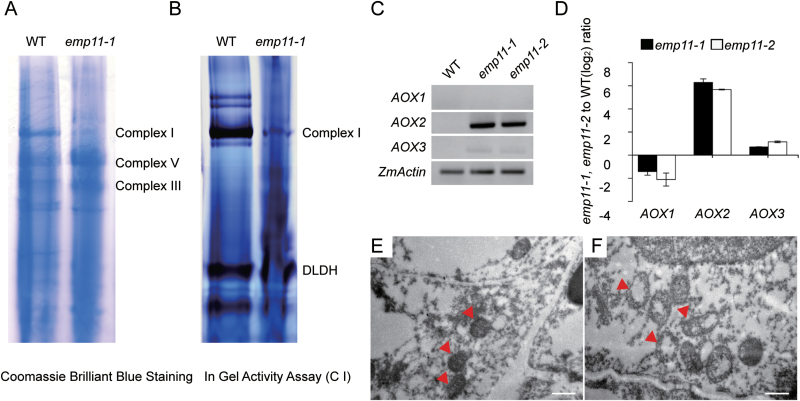
Mitochondrial structure and respiratory function in *emp11* mutants. (A) BN gels were stained with Coomassie Brilliant blue. Mitochondrial complexes of the embryo and endosperm of maize kernels at 12 DAP were subjected to a 3% to 12% BN-PAGE. (B) In-gel NADH dehydrogenase activity assay of the mitochondrial protein complexes was assayed using dihydrolipoamide dehydrogenase (DLDH) as a loading control. (C) The transcript levels of *AOX1*, *AOX2*, and *AOX3* in *emp11-1* and *emp11-2* kernels. Total RNA was extracted from 15 DAP kernels with the pericarp removed. The expression levels were normalized to *ZmActin* (GRMZM2G126010). (D) qRT-PCR analysis of *AOX1*, *AOX2*, and *AOX3* in wild-type, *emp11-1*, and *emp11-2* plants. (E, F) Transmission electron micrographs of ultra-thin sections from the endosperm of wild-type (E) and *emp11* mutants (F). Mitochondria are indicated with arrows. Scale bars, 0.5 µm.

Nad1 is a central component of complex I and is essential for assembly of the complex in the membrane (Klodmann *et al.*, 2010). Substantial reduction in *nad1* expression is likely to impact complex assembly, stability, and/or activity. To uncover whether the defect in *nad1* processing in *emp11* mutants affected complex I formation and/or function, we examined complex I assembly with Blue Native polyacrylamide gel electrophoresis (BN-PAGE) and complex I activity with an in-gel NADH dehydrogenase activity assay. The results showed strongly reduced assembly and activity of complex I in *emp11* mutants (Fig. 5A, B), indicating that the loss of *Emp11* expression led to a reduction in the assembly and activity of complex I.

To further investigate the consequences of the *emp11* mutation, we analyzed the alternative pathway by examining the expression of three alternative oxidases, *AOX1* (AY059646.1), *AOX2* (AY059647.1), and *AOX3* (AY059648.1). We found that *AOX2* expression was dramatically increased in *emp11* mutants and *AOX3* expression was also higher (Fig. 5C, D). These results indicate that the loss of *Emp11* function causes a dramatic increase in the expression of *AOX* genes.

Reduced complex I levels in Arabidopsis *nmat1* and *nmat4* mutants are considered to contribute to the compromised mitochondrial ultrastructure (Keren *et al.*, 2012; Sosso *et al.*, 2012; Cohen *et al.*, 2014). To observe the ultrastructure of *emp11* mitochondria, we used ultra-thin sections of 10 DAP endosperm for TEM and found that wild-type mitochondria showed normal cristae, visible as densely folded inner membranes (Fig. 5E), while approximately 40% of *emp11-1* mitochondria had a poorly developed membrane system with a large internal space and abnormal and obscure cristae (Fig. 5F). Based on the mitochondrial ultrastructure, we assume that the respiratory function of the mutant mitochondria may be impaired.

### The phenotype of *emp11* is modified in B73 BC_2_F_2_ segregating ears

In BC_2_F_2_ segregating ears in B73, we observed partially filled *emp11-1/emp11-1* kernels with weights approximately one fifth of that of wild-type, larger than in W22 (Supplementary Fig. S7A, B). Furthermore, we found that *emp11-1* (B73), *emp11-1* homozygous kernels in BC_2_F_2_ segregating ears in B73, embryos were larger than in W22 with thick scutellum, small coleorhiza, short roots, stunted coleoptile, and abnormal shoot apex lacking clear differentiation (Supplementary Fig. S7C). Moreover, homozygous *emp11-1* (B73) seeds had a 55% germination ratio, but these seedlings exhibited low a survival rate of ~23%) and the survived seedlings had much smaller roots and shoots relative to wild-type(B73) (Supplementary Fig. S7D, E). We measured agronomically important traits, including plant height, ear height, kernel row number, leaf width, spikelet density, tassel branches, and tassel length of those surviving seedlings, and found that these traits were severely arrested in *emp11-1* (B73) plants (Supplementary Fig. S7F–L).

## Discussion

### *Emp11* affects complex I by modulating the intron splicing of *nad1* in maize mitochondria

NAD1 is a central component of complex I, which is located on the mitochondrial membrane (Klodmann *et al.*, 2010; Meyer *et al.*, 2011). NAD1 deficiency leads to disturbances in the assembly or stability of complex I (Keren *et al.*, 2009; Keren *et al.*, 2012; Cohen *et al.*, 2014). In maize, Dek2, a mitochondrial P-type PPR protein, is involved in the splicing of *nad1* intron 1 (Qi *et al.*, 2016). In Arabidopsis, there are at least three maturases and various other protein cofactors involved in the maturation of *nad1* (Cohen *et al.*, 2014), including OTP43, a P-type PPR protein, which is specifically required for *trans*-splicing of mitochondrial *nad1* intron 1 and *cis*-splicing of *nad2* intron 1 (Falcon de Longevialle *et al.*, 2007). In our study, the four introns within *nad1* pre-mRNA could not be removed normally because of the loss of *Emp11* function, while other mitochondrial introns in *emp11* were correctly spliced, suggesting that a single *emp11* mutation affects the splicing of all the introns of *nad1*. It is well known that the mechanism of mitochondrial intron splicing is a very complex procedure involving many splicing factors, which facilitate the splicing of group II introns, including PPR proteins, CRM (chloroplast RNA splicing and ribosome maturation) proteins, RNA DEAD-box helicases, PORR (plant orangellar RNA recognition) domain proteins, RCC (regulator of chromosome condensation) proteins, and others. Among them, PPR, PORR, and RCC may act as the factors that recognize the specific RNA-binding sites (Brown *et al.*, 2014). To interpret the non-specificity of EMP11 to *nad1* introns, we assume that EMP11 may play a role in the recognition of precursor *nad1* mRNA and in the maintenance of the *nad1* conformation for intron splicing by cooperating with other factors. The absence of mature *nad1* transcripts led to the failure of complex I assembly (Fig. 5). This is consistent with early reports in complex I-deficient *nmat1* (Keren *et al.*, 2012), *nmat2* (Keren *et al.*, 2009), and *nmat4* (Cohen *et al.*, 2014) mutants. Nucleus-encoded factors play essential roles in the regulation of mitochondrion development, which requires the coordinated expression of both nucleus-encoded and mitochondrion-encoded genes, such as *ppr8522*, a plastid mutant that triggers a retrograde signal to the nucleus (Sosso *et al.*, 2012). These results support the existence of a retrograde signaling pathway coordinating mitochondrial and nuclear gene expression. This retrograde signaling may be associated with the mitochondrial intron splicing machinery to monitor its integrity. In our study, *Dek2* and some orthologs of the Arabidopsis *nad1* splicing genes, *OTP43*, *nMAT1*, *nMAT2*, *nMAT4*, *mCSF1*, and *PMH2*, had higher transcript levels in *emp11* mutants. We propose that *nad1* splicing in maize likely shares a similar mechanism as that in Arabidopsis and the absence of *Emp11* induces a higher expression of *nad1* splicing-related genes in the *emp11-1* mutant. The discrepancies in the splicing efficiencies of *nad1* introns and up-regulated expression of other *nad1* splicing genes in *emp11* mutants suggest that *Emp11* possibly influences a plethora of metabolic and developmental changes.

ATP production within mitochondria is linked to electron transfer through complex I (Millar *et al.*, 2011). Defects in complex I likely lead to loss of ATP synthesis, alter signal transduction pathways, and disturb normal metabolism (Schmitz-Linneweber and Small, 2008). In our study, the mitochondrial structure of *emp11* mutants was altered (Fig. 5F). This altered mitochondrial morphology is likely unable to provide enough ATP to maintain normal life activities. Under this low energy condition, the alternative respiratory pathway is activated (Gutierres *et al.*, 1997; Sabar *et al.*, 2000; Karpova *et al.*, 2002; Dutilleul *et al.*, 2003). Our data showed that *AOX2* transcription levels were dramatically increased in *emp11* mutants (Fig. 5C, D), suggesting that the AOX pathway was activated by the loss of *Emp11* expression. Complex I mutants are anticipated to display retarded phenotypes with empty pericarp seeds, which may result from deficits in energy supply. For example, in Arabidopsis*, otp43*, *nmat1*, and *nmat4* mutants display similar growth defects as they all fail to process *nad1* transcripts correctly and show altered respiratory function as a consequence of complex I defects (Falcon de Longevialle *et al.*, 2007; Keren *et al.*, 2012; Cohen *et al.*, 2014). Taken together, these data suggest that EMP11 serves as a factor involved in the splicing of mitochondrial *nad1* introns and affects the respiratory function of complex I, which is the physiological basis for ensuring normal embryo and endosperm development in maize kernels.

### *Emp11* is required for seed and plant development and the *emp* phenotype could be partially recovered in B73 BC_2_F_2_ segregating ears

The maize embryo and endosperm are two products of double fertilization. Endosperm development includes coenocytic, cellularization, differentiation, and maturation stages. The differentiated endosperm contains four major cell types: the cells of the embryo surrounding region, the BETL, the aleurone layer, and the starchy endosperm (Olsen, 2001). The BETL is responsible for nutrient transfer from maternal tissue to the developing endosperm (Olsen, 2004). mRNA *in situ* hybridization revealed that *Emp11* was highly expressed in BETL cells (Fig. 3D), suggesting that *Emp11* is important for BETL development. This inference was then supported by the fact that BETL was absent from *emp11* kernels at 15 DAP (Fig. 1J) and was also further supported by the observation that the starchy endosperm was poorly filled in *emp11* mutants (Fig. 1F and Supplementary Fig. S1J). Embryo development in maize proceeds in three stages, namely the transition, coleoptilar, and late embryogenesis stages (Olsen, 2001). In this study, we found that *Emp11* was highly expressed in embryos (Fig. 3A). In the W22 background, the loss of *Emp11* function caused the early arrest of embryo development at the transition stage (Fig. 1 and Supplementary Fig. S1). Homozygous *emp11-1* (B73) embryos could sometimes develop and germinate into plants, but they had delayed development relative to wild-type (B73) plants (Fig. 6E), suggesting that EMP11 also acts during post-embryonic development. Background-dependence has also been described for *ppr8522* and *whirly1*, two plastid-targeted PPR genes that confer an *embryo-specific* (*emb*) phenotype (Prikrylv *et al.*, 2008; Sosso *et al.*, 2012; Zhang *et al.*, 2013). In the *R-scm2* background, the *emb8522* mutant causes embryo lethality but causes *albino* seedlings in the A188 and B73 backgrounds (Sosso *et al.*, 2012). In addition, the *why1* allele in the W22 genetic background causes specific arrest in embryogenesis without a major impact on endosperm development. However, the severe phenotype can be suppressed in maize A188, B73, Mo17 and Oh51a genetic backgrounds, giving rise to seedlings, which show an albino seedling phenotype (Prikrylv *et al.*, 2008; Zhang *et al.*, 2013). The retrograde signaling hypothesis is used to explain the background-dependent suppression of *why1* in embryogenesis (Zhang *et al.*, 2013). The *emp11* phenotype maybe background-dependent and there appears to be suppressor(s) in B73 that can suppress the *emp* phenotype of *emp11*, as we observed that homozygous *emp11* seeds in B73 BC_2_F_2_ segregating ears have the totipotency to develop a plant. A similar finding was reported by introgresing single *emp* mutants in different genetic backgrounds and revealed the existence of a cryptic genetic variation (CGV) recognizable as a variable increase in endosperm tissue (Sangiorgio *et al.*, 2016). The genetic difference between B73 and W22 may provide clues to understanding the background dependence of the *emp11* phenotype, which showed embyro lethality under the W22 background but embryo totipotency in B73 BC_2_F_2_ segregating ears.

## Supplementary data

Supplementary data can be found at *JXB* online.

Table S1. Primers used in this study.

Fig. S1. Embryo and endosperm development in early stage. 

Fig. S2. Genotype and phenotype analysis of *emp11* mutants alleles. 

Fig. S3. Defective kernels were produced in *Emp11* RNAi transgenic lines. 

Fig. S4. Alignment of the EMP11 protein with rice ortholog LOC_Os11g04295, sorghum ortholog Sb05g002210 and Arabidopsis ortholog AT1G52620. 

Fig. S5. Expression of *Emp11* in Multiple Organs.

Fig. S6. *Emp11* is specifically involved in *nad1* introns splicing. 

Fig. S7. The *emp* phenotype of the *emp11-1* mutants can be suppressed in BC_2_F_2_ of B73. 

## Author contributions

Conceived and designed the experiments: ZZ FQ XR ZP. Performed the experiments: XR ZP HZ. Analyzed the data: XR ZP HZ. Contributed reagents/materials/analysis tools: JZ MC. Wrote the manuscript: XR ZP ZZ FQ.

## Supplementary Material

supplementary_figures_S1_S7_Table_S1

## Abbreviations:

AOX: alternative oxidase
BETL: basal endosperm transfer layer
BN-PAGE: blue native PAGE
DAP: days after pollination
EMP11: *empty pericarp 11*
NB: normal type in nuclear background B73
nMat: , nuclear maturases
MATs: maturases
PPR: pentatricopeptide repeat.
