# Supplementary material for: EMPTY PERICARP11 serves as a factor for splicing of mitochondrial nad1 intron and is required to ensure proper seed development in maize
Source: J Exp Bot. 2017 Sep 30;68(16):4571–81. doi: 10.1093/jxb/erx212 (PMC5853838; doi:10.1093/jxb/erx212)
Supplement: supplementary_figures_S1_S7_Table_S1 [file erx212_suppl_supplementary_figures_s1_s7_table_s1.pdf]

**The P-type Pentatricopeptide Repeat Protein EMPTY PERICARP11 is Required for Mitochondrial *nad1* Intron Splicing and Seed Development in Maize**

## Supplementary data

Supplementary data can be found at JXB online.

There are one supplementary table and seven supplementary figures in this file, including Supplementary Table S1 and Supplementary Fig. S1-Supplementary Fig. S7.

**Table S1.** Primers used in this study.

| Primer name | Primer sequence | Use |
|---|---|---|
| 08197F | GCCGTTTTCTACAATGTGCTC | Genotype test for UFMu-08197 |
| 08197R | GTAACCGCCTCTTTCATCATTC | |
| 04323F | ACTGCAACATCGGTGACACA | Genotype test for UFMu-04323 |
| 04323R | CCACCGGAAACTTTGCCTTG | |
| R565 | CATACGTAACCACGGTTGGCATGA | 5'RACE |
| R468 | GAGCACATTGTAGAAAACGGCACCC | |
| F69 | GCTGCGGACCCCATACCGAGCTTCTGAC | 3'RACE |
| F206 | CAGGTACGCGAATTAAATCAGCCGCTCC | |
| Emp11-Kpn1-F | GGTACC ATGTCCCTCGCGGGGGCCGCGC | Subcellular location |
| Emp11-Xba1-R | GTTCTAGAGGAACCAGCGAATCTCTGCtct | |
| RT-Emp11-3F | AGATGTCGTCACGTTTAACACT | In situ hybridization and RT |
| RT-Emp11-3R | CACTCCTAACGAACCCATCAAT | |
| qEmp11-F1 | GGATGTTCGAGAGGCTCTGA | Real-time quantitative PCR |
| qEmp11-R1 | GAGTATGCCCTCTCCCCATC | |
| GM301-1F | CTAGTATCCCGGGAAGGCGCGCCTCAGGAACCAG CGAATCTCTGCTCT | Emp11 antisense for RNAi analysis |
| GM301-1R | GAACGATAAGCTTATGGCGCGCCATGTCCCTCGCG GGGGCCG | |
| nMAT1-3938F2 | CATCAGCTGGGAAGGAGAAG | RT-PCR |
| nMAT1-3938R2 | CCGCGTAGAGCTTTGCTAGT | |
| OTP43-6058F2 | AGAAGGGCAACTTGGAGGAT | RT-PCR |
| OTP43-6058R2 | GCAAGCGCGTATATTTCTCC | |
| PMH2-0512F2 | CCTGGGGTTTAGGTCCACTC | RT-PCR |
| PMH2-0512R2 | AGCCAGCATTTGATCAGCTT | |
| mCSF-7395F2 | GACCGTTACGGCTTCGTG | RT-PCR |
| mCSF-7395R2 | GTTGTGAACGTCGCTTAGCA | |
| PMH2-7984F2 | ACAACGGGTCGTTCTACGAG | RT-PCR |
| PMH2-7984R2 | AGGTGTCCCAATGACCACAT | |
| nMAT2-4119F2 | TCCATTGCCTATCCTCATCC | RT-PCR |
| nMAT2-4119R2 | CGAGAACCTGGCTTCGTAGA | |
| nMAT4-5999F2 | CGGTAGGGAGCACCGTAGG | RT-PCR |
| nMAT4-5999R2 | CGGAAAAACAGAGGCGTTAG | |
| PMH2-5140F2 | AGTGGACCTTGTTGGTGAGG | RT-PCR |
| PMH2-5140R2 | GGCCAGATCTGTGAACGAAT | |
| Dek2-RT-F | GAGGTTGACAGGGCATTGAT | RT-PCR |
| Dek2-RT-R | CATTGCCCGAGGTAAGTCAT | |
| TransDNA-F | CTCCCAAGCCAGTGAATGAT | Genotype test for RNAi |
| TransDNA-R | TTGCGCGCTATATTTTGTTTT | |
| TransRNA-F | CCAGCAGCACTGTTGAAACT | Real-time quantitative PCR for RNAi |
| TransRNA-R | TTGCGCTAGATGCTAACACG | |

**Fig. S1**

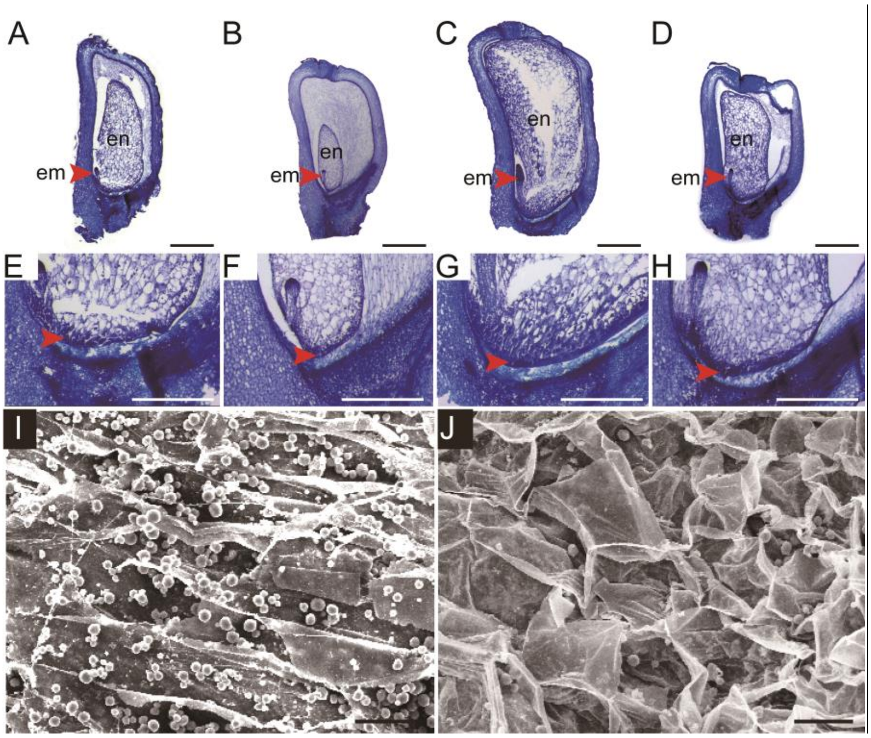


**Fig. S1.** Embryo and endosperm development in early stage.

(A)-(H) Sections of developmental kernels at 6-DAP (A-B, E-F) and 8-DAP(C-D, G-H). WT (A, C) and *emp11-1* kernels (B, D). (E-H) are magnified images of the micrographs above. Arrows indicate the BETL. em, embryo; en, endosperm. (I-J) Starch accumulation in the kernels of WT(I) and *emp11-1*(J) at 15-DAP. Scale bar = 1mm in (A-D), 500 μm in (G-H) and 200 μm in (I-J).

**Fig. S2**

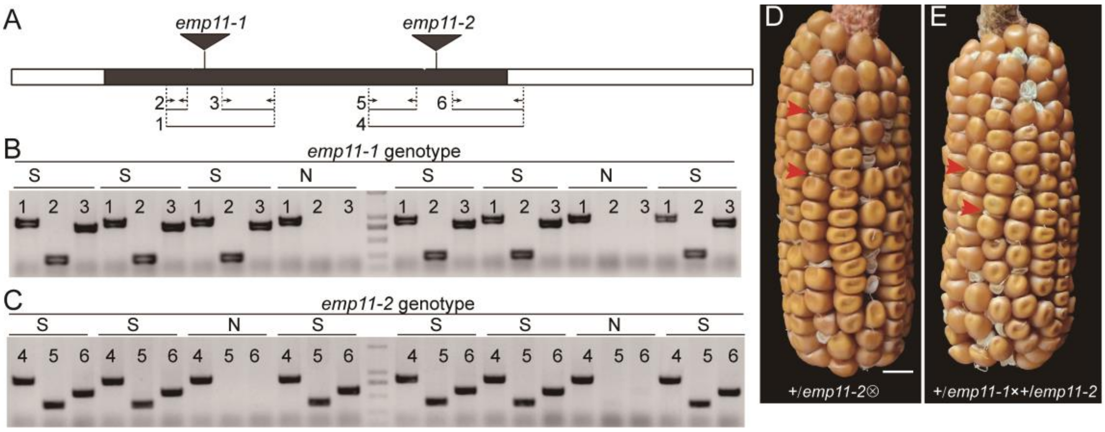


**Fig. S2.** Genotype and phenotype analysis of *emp11* mutants alelles.

(A) Gene structure of *Emp11* and locations of the *Mu* insertions in two independent alleles. The primers were indicated with arrows. (B-C) Genotype analysis of *emp11* mutants alelles. 08197F/R with TIR6 for *emp11-1*(B) and 04323F/R with TIR6 for *emp11-2*(C). N, nonsegregating (the wild type, genotype is +/+ ); S, segregating (heterozygous, genotype is +/*emp11-1* or +/*emp11-2*). (D) Phenotype of self-pollinated +/*emp11-2* segregating ear. The arrow identifies the *emp11-2* mutant seeds. (E) Allelic tests between +/*emp11-1* and +/*emp11-2* mutants. The generated ear from +/*emp11-1* ×+/*emp11-2*. The arrow identifies the seed of mutant phenotype. Scale bar=1 cm.

**Fig. S3**

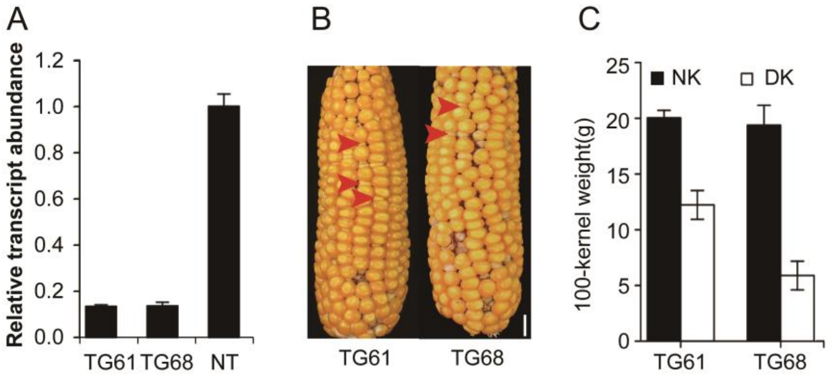


**Fig. S3. Fig. S3. Defective kernels were produced in *Emp11* RNAi transgenic lines.**

**Fig. S3. Defective kernels were produced in *Emp11* RNAi transgenic lines.**

(A) *Emp11* expression in the $T_0$ progeny of RNAi transgenic lines TG61 and TG68. The RNA was isolated from the leaves of the RNAi transgenic and NT plants. The expression levels were normalized to *ZmActin* (GRMZM2G126010). NT, non-transgenic line. (B) Segregated ears in the $T_1$ progeny of RNAi transgenic lines TG61 and TG68. Scale bar = 1 cm. (C) The 100-weight of the normal and defective kernels(DK) in TG61 and TG68. Arrows show the defective kernels.

**Fig. S4**

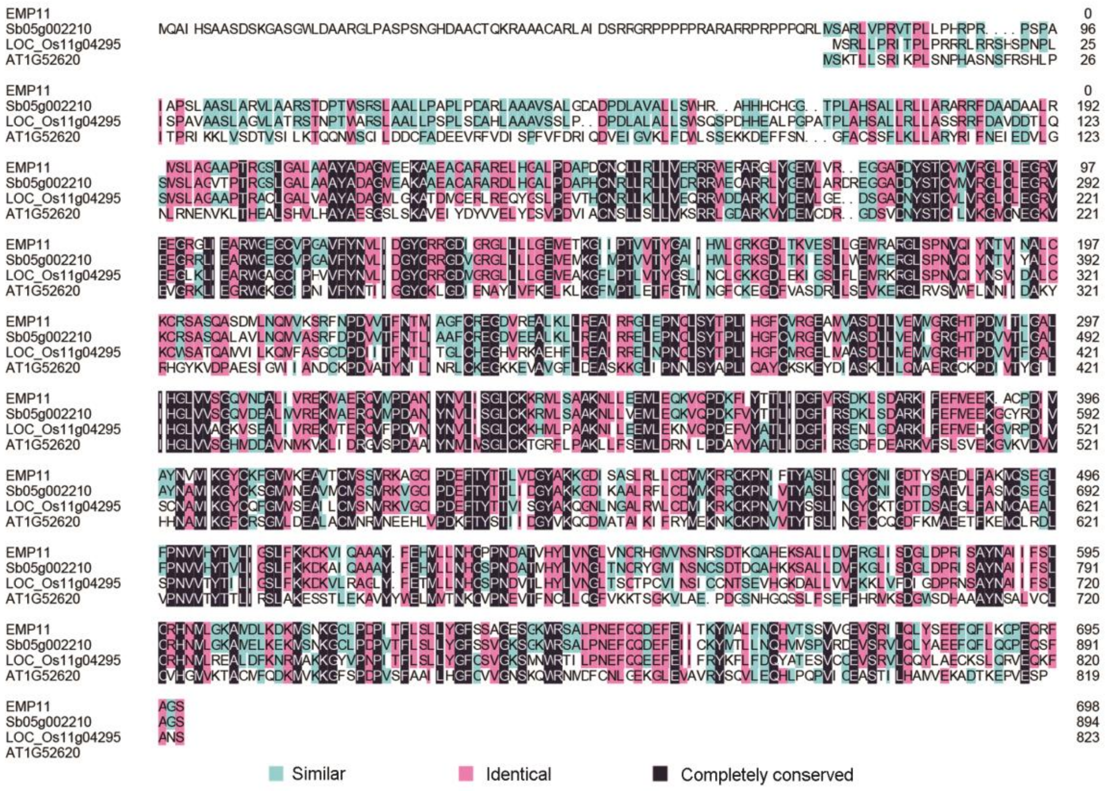

**Fig. S4.** Alignment of the EMP11 protein with rice ortholog LOC_Os11g04295, sorghum ortholog Sb05g002210 and Arabidopsis ortholog AT1G52620.

**Fig. S5**

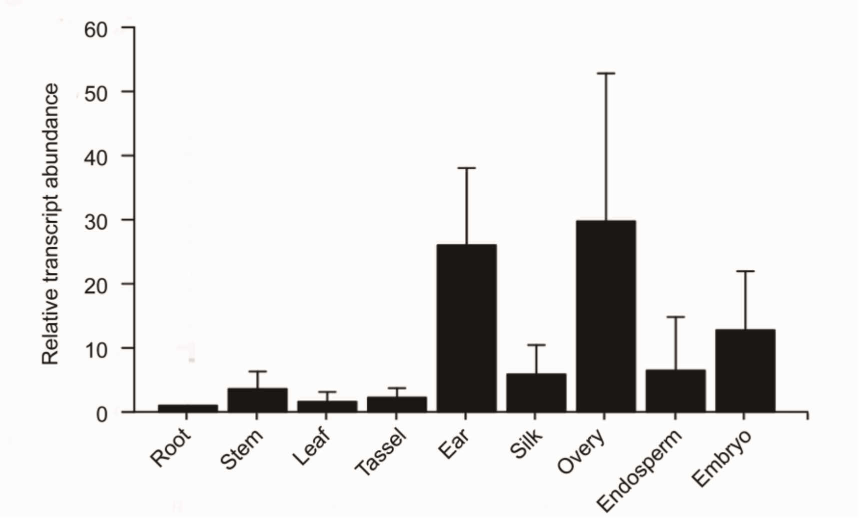


**Fig. S5.** Expression of *Emp11* in Multiple Organs.

**Fig. S6**

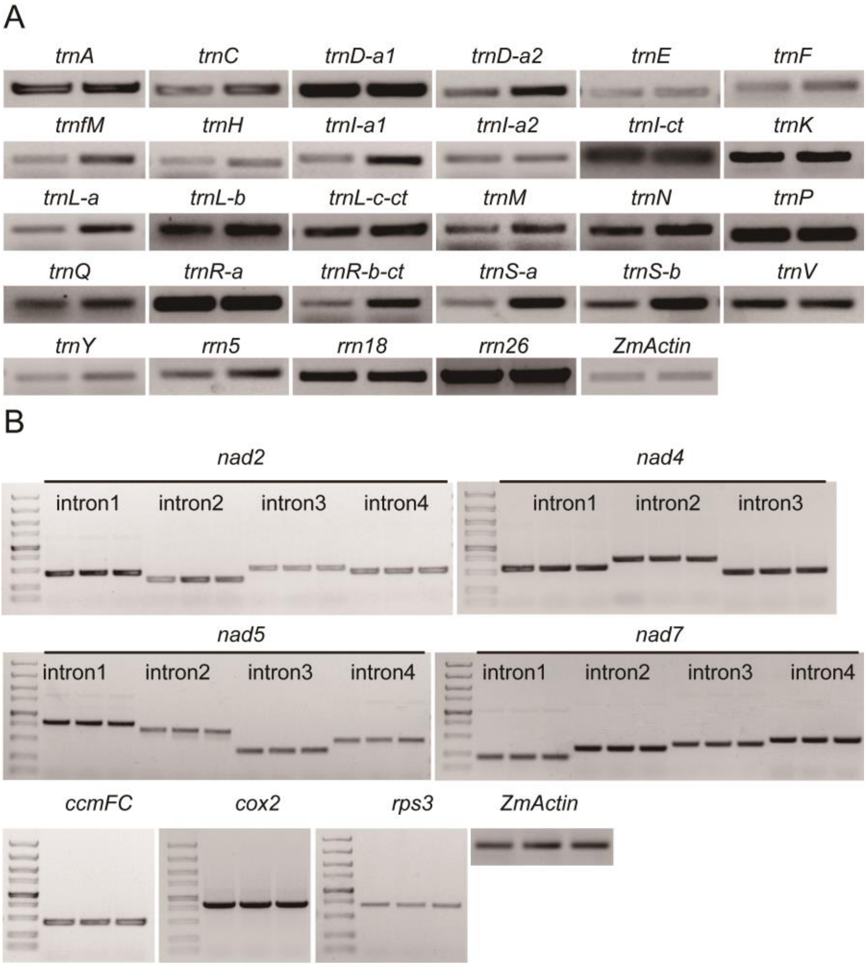


**Fig. S6. *Emp11* is specifically involved in *nad1* introns splicing.**

(A) RT-PCR analysis of mitochondrial tRNA and rRNA encoded genes transcriptional levels in the wild type and the *emp11-1* mutant. In each of the gels, the left lane is wild-type and the right lane is *emp11-1* mutant.

(B) RT-PCR analysis of all 22 group II introns in maize mitochondrial genes was performed with RNA isolated from two mutant alleles and wild-type (WT) kernels. In each gel of the introns, the three lanes are WT, *emp11-1* and *emp11-2* mutant, respectively.

**Fig. S7**

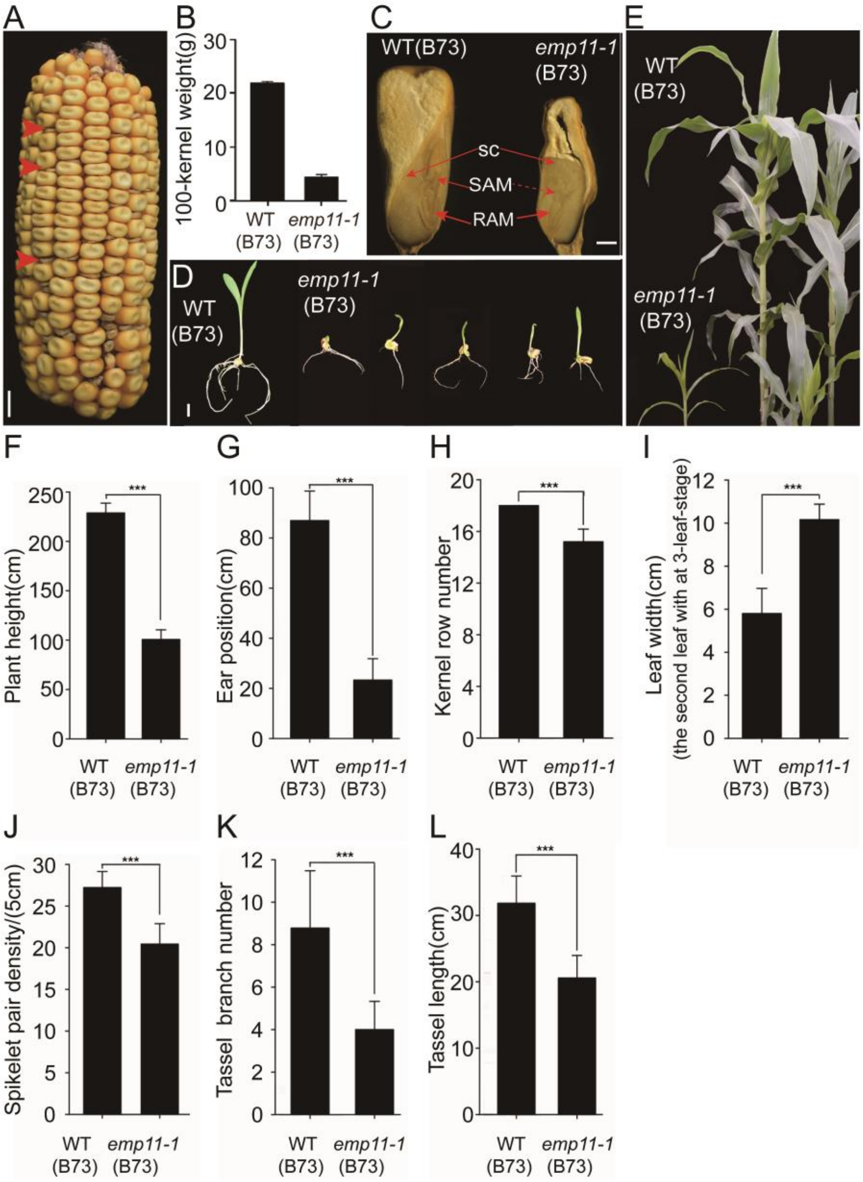


**Fig. S7. The *emp* phenotype of the *emp11-1* mutants can be suppressed in $BC_2F_2$ of B73.**

(A) The self-pollinated *emp11-1* heterozygotes in $BC_2F_2$ of B73. The mutant kernels are indicated by arrows. (B) The 100-kernel weight of WT and *emp11-1* mutants in $BC_2F_2$ of B73. (C) Germinal side view of the mature WT and *emp11-1* kernel in $BC_2F_2$ of B73. (D) The seedlings of WT and *emp11-1* in $BC_2F_2$ of B73. (E) The plants of WT and survived *emp11-1* in $BC_2F_2$ of B73. Scale bar = 1 cm in (A) and (D), and 1

mm in (C). (F-L) The agronomically important traits of WT(B73) and *emp11-1*(B73).
